# A simple, inexpensive method for preparing cell lysates suitable for downstream reverse transcription quantitative PCR

**DOI:** 10.1038/srep04659

**Published:** 2014-04-11

**Authors:** Kenneth Shatzkes, Belete Teferedegne, Haruhiko Murata

**Affiliations:** 1 Laboratory of DNA Viruses, Division of Viral Products, OVRR, CBER, FDA, Bethesda, MD 20892, USA; 2 Laboratory of Pediatric and Respiratory Viral Diseases, Division of Viral Products, OVRR, CBER, FDA, Bethesda, MD 20892, USA; 3 These authors contributed equally to this work.; 4 Current address: Graduate School of Biomedical Sciences, New Jersey Medical School and Rutgers School of Dental Medicine, Newark, New Jersey, USA.

## Abstract

Sample nucleic acid purification can often be rate-limiting for conventional quantitative PCR (qPCR) workflows. We recently developed high-throughput virus microneutralization assays using an endpoint assessment approach based on reverse transcription qPCR (RT-qPCR). The need for cumbersome RNA purification is circumvented in our assays by making use of a commercial reagent that can easily generate crude cell lysates amenable to direct analysis by one-step RT-qPCR. In the present study, we demonstrate that a simple buffer containing a non-ionic detergent can serve as an inexpensive alternative to commercially available reagents for the purpose of generating RT-qPCR-ready cell lysates from MDCK cells infected with influenza virus. We have found that addition of exogenous RNase inhibitor as a buffer component is not essential in order to maintain RNA integrity, even following stress at 37°C incubation for 1–2 hours, in cell-lysate samples either freshly prepared or previously stored frozen at −80°C.

Quantitative PCR (qPCR) is associated with several appealing performance features such as its sensitivity (which can allow quantification of targets approaching the limiting concentration in molecular terms) and its dynamic range (which can span several orders of magnitude). Despite these obvious advantages, full realization of the potential of qPCR has been hindered, particularly for high-throughput applications, because sample nucleic acid purification required in a conventional workflow can often be cumbersome and rate-limiting. We recently developed microneutralization assays for influenza virus^1^ and respiratory syncytial virus^2^ with endpoint assessment based on reverse transcription qPCR (RT-qPCR) that uses samples generated by a procedure that circumvents the need for RNA purification. In our assays, virus-infected cells (in a 96-well plate format) are washed and then briefly exposed to a commercially available cell-lysis reagent; the resulting cell lysates are subjected to direct analysis by one-step RT-qPCR in order to measure the expression level of a viral gene target. Samples prepared in this straightforward manner require minimal effort. Thus, our approach might be suitable even for large-scale serological studies.

Commercial reagents for the generation of RT-qPCR-ready cell lysates have now become available from several sources. These reagents have garnered increasing interest as tools for enabling high-throughput gene-expression analysis^3^,^4^. Recent studies have validated the accuracy of RT-qPCR relying on commercial cell-lysis reagents^5^,^6^, providing justification and incentive for expanded use. Despite the appeal of simplicity afforded by these commercial cell-lysis reagents, the attendant high cost can be problematic. In addition, the components of these proprietary reagents are undisclosed, which can limit experimental flexibility for the end user. In the present study, we sought to develop an inexpensive alternative to commercial reagents. We hereby demonstrate that a simple buffer containing a non-ionic detergent can generate cell lysates for use in our RT-qPCR-based influenza virus microneutralization assay. Surprisingly, we have found that addition of exogenous RNase inhibitor as a buffer component is not obligatory to maintain sample RNA integrity. Avoidance of exogenous RNase inhibitor addition allows per-sample cost of generating cell lysates for RT-qPCR to be essentially negligible using our buffer.

## Results

### Optimization of buffer formulation for the preparation of cell lysates

**Figure 1:**
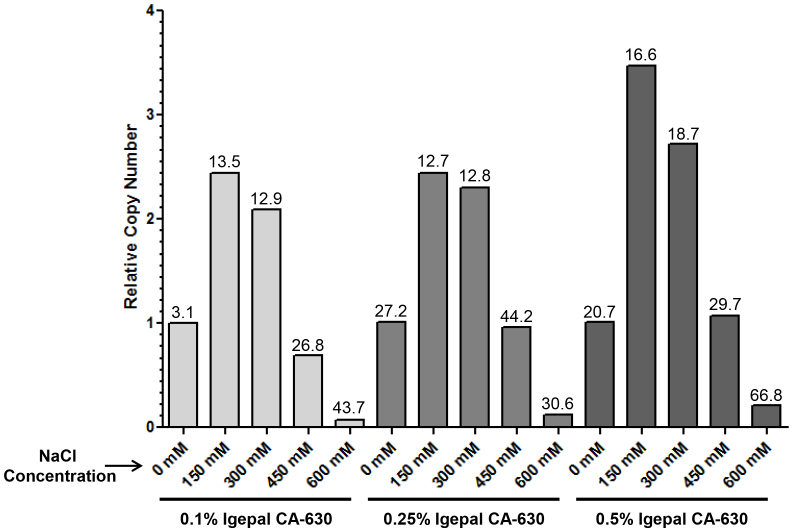
Optimization of the concentration of Igepal CA-630 and NaCl. MDCK-London cells (300,000 cells per well of a 24-well plate) were infected with influenza virus (A/Brisbane/59/2007; 10,000 TCID_50_ per well). Six hours post-infection, cell monolayers were washed once with phosphate-buffered saline (PBS) and then exposed to 200 μL of the appropriate lysis buffer [10 mM Tris-HCl pH 7.4, Igepal CA-630 (0.1, 0.25, or 0.5%), and NaCl (0, 150, 300, 450, or 600 mM)] for 2 minutes at room temperature. The resulting cell lysates were carefully collected, and 1 μL of each sample was analyzed directly by one-step SYBR Green RT-qPCR (10 μL total reaction volume) with primers targeting the influenza virus matrix gene. For each buffer condition, results were normalized to the mean value obtained with buffer containing 0.1% Igepal CA-630 and 0 mM NaCl; mean relative copy numbers are shown along with % relative standard deviation above each bar (calculated from two independent experiments, each with three RT-qPCR replicates per condition; n = 6).

In our attempt to develop a cell-lysis reagent suitable for preparing samples to be used in downstream RT-qPCR, we were guided by established protocols describing the extraction of cytoplasmic RNA from cultured cells^7^,^8^; generally, these protocols involve exposing cells to a lysis buffer containing a non-ionic detergent, followed by a purification procedure for RNA (*e.g*., extraction with phenol/chloroform) applied to the cleared supernatant resulting from the cell lysis step. We initially evaluated lysis-buffer formulations with a limited number of components to minimize the risk of detrimental impact on downstream reverse transcription and PCR. Buffers containing 10 mM Tris-HCl pH 7.4, Igepal CA-630 (0.1, 0.25, or 0.5%), and NaCl (0, 150, 300, 450, or 600 mM) were prepared from stock solutions on the day of experimentation and equilibrated to room temperature (approximately 22°C) prior to use. Experimental conditions were designed to parallel (although modestly upscaled to a format using 24-well culture plates rather than 96-well culture plates to ensure sufficient sample quantity for analysis) those described for our RT-qPCR-based influenza virus microneutralization assay^1^. MDCK-London cells (300,000 cells per well of a 24-well plate) were infected with influenza virus (A/Brisbane/59/2007; 10,000 TCID_50_ per well). Six hours post-infection, cell monolayers were washed once with phosphate-buffered saline (PBS) and then exposed to 200 μL of the appropriate lysis buffer for 2 minutes at room temperature. The resulting cell lysates were carefully collected, and 1 μL of each sample was analyzed directly by one-step SYBR Green RT-qPCR (10 μL total reaction volume) with primers targeting the influenza virus matrix gene. Data for these experiments are summarized in Fig. 1. For each buffer condition, results were normalized to the mean value obtained with buffer containing 0.1% Igepal CA-630 and 0 mM NaCl; mean relative copy numbers are shown along with % relative standard deviation (calculated from two independent experiments, each with three RT-qPCR replicates per condition; n = 6). Relative copy numbers were maximized (*i.e*., C_q_ values were minimized) at 150 mM NaCl regardless of Igepal CA-630 concentration. Either in the absence of NaCl or at higher concentrations (300–600 mM), relative copy numbers decreased, suggesting reduced cell lysate RNA yield and/or suboptimal downstream RT-qPCR. An increase in data variability was also observed at the highest NaCl concentration tested (600 mM). Similar results were obtained with lysis buffers containing Triton X-100 instead of Igepal CA-630 (data not shown).

Other buffer parameters were evaluated in isolation using a similar experimental approach involving MDCK-London cells infected with influenza virus. Protocols for extraction of cytoplasmic RNA often use a lysis buffer containing MgCl_2_^7^,^8^. We explored the impact of the absence or presence of MgCl_2_ (1.5 or 5 mM) in a buffer containing 10 mM Tris pH 7.4, 0.25% Igepal CA-630, and 150 mM NaCl. The presence of MgCl_2_ at millimolar concentrations resulted in modest increases in C_q_ values (ΔC_q_ ranging from 1.18 to 2.44), again possibly indicating reduced RNA recovery and/or impairment of downstream RT-qPCR (Supplementary Table 1). In addition, we evaluated the effect of pH (10 mM Tris pH 7.0, 7.4, or 8.0; 0.25% Igepal CA-630; 150 mM NaCl). Within the range tested, we found the pH of the lysis buffer to have minimal influence on C_q_ values (Supplementary Table 2). For subsequent experiments, we chose to proceed with a buffer containing 10 mM Tris pH 7.4, 0.25% Igepal CA-630, and 150 mM NaCl, which we refer to hereafter as Cell-Lysis (CL) Buffer.

**Figure 2:**
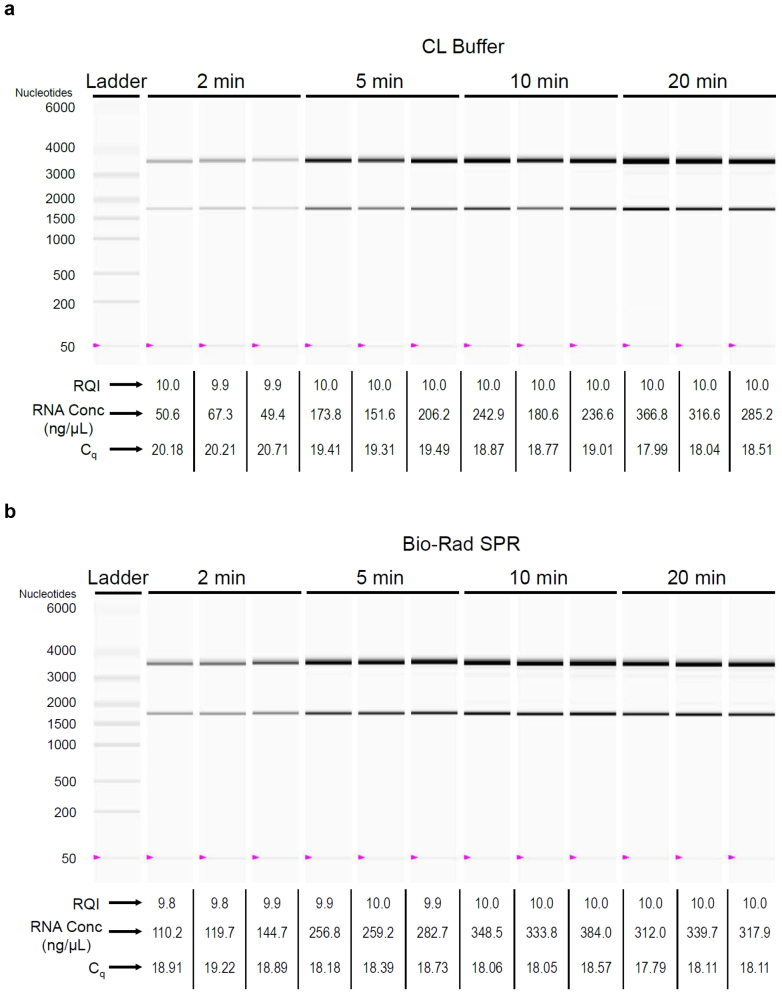
Duration of cell exposure to cell-lysis reagent. Cell lysates (200 μL) were prepared from MDCK-London cells (300,000/well; 24-well plate) infected with influenza virus (10,000 TCID_50_/well) by exposing them to (a) CL Buffer or (b) Bio-Rad SPR for 2–20 min at ~22°C. One μL of each resulting lysate was analyzed directly by one-step SYBR Green RT-qPCR with primers targeting the influenza virus matrix gene; total RNA was purified immediately from the remaining lysates and subjected to microfluidics-based electrophoresis using the Bio-Rad Experion system. Virtual gel images, sample RNA yields, and RNA Quality Indicators (RQIs) from a representative experiment (all derived from the Experion analysis) are shown; associated C_q_ values from RT-qPCR are also indicated. For each condition, three independently prepared cell lysates were evaluated.

We next assessed the duration of cell exposure to CL Buffer. MDCK-London cells (24-well plate; 300,000 cells per well) were infected with influenza virus (A/Brisbane/59/2007; 10,000 TCID_50_ per well); six hours post-infection, cell monolayers were washed once with PBS and then exposed to 200 μL of CL Buffer or the Bio-Rad iScript Sample Preparation Reagent (subsequently referred to as Bio-Rad SPR) for 2 to 20 minutes at room temperature. One μL of each resulting lysate was analyzed directly by one-step SYBR Green RT-qPCR with primers targeting the influenza virus matrix gene; total RNA was purified immediately from the remaining lysates using Qiagen RNeasy columns and subjected to microfluidics-based electrophoresis using the Bio-Rad Experion Automated Electrophoresis system^9^. Virtual gel images, sample RNA yields, and RNA Quality Indicators (RQIs; ranging from 1.0 for highly degraded RNA to 10.0 for intact RNA) from a representative experiment (all derived from the Experion analysis) are shown in Fig. 2; associated C_q_ values from RT-qPCR are also indicated. For each condition, three independently prepared cell lysates were evaluated. Total RNA was generally intact (RQIs of 9.9–10.0) in lysates freshly prepared using CL Buffer (Fig. 2a); two distinct major bands on the virtual gel representing 28S and 18S ribosomal RNA were observed at a ratio close to the expected value (1.7–1.9). Mean RNA concentration of purified samples increased from 55.8 ng/μL at 2 minutes of cell exposure to 177.2 ng/μL at 5 minutes of cell exposure; by 20 minutes, mean RNA concentration was 322.9 ng/μL. Mean C_q_ values (n = 3) reflected this dynamic, decreasing from 20.37 at 2 minutes of cell exposure to 18.18 at 20 minutes of cell exposure. Similarly, total RNA was intact (RQIs of 9.8–10.0) in lysates generated using Bio-Rad SPR (Fig. 2b). Mean RNA concentration of purified samples generated with Bio-Rad SPR increased from 124.9 ng/μL at 2 minutes of cell exposure (associated mean C_q_ value of 19.01) and approached a plateau at 20 minutes of cell exposure (323.2 ng/μL; associated mean C_q_ value of 18.00). On the basis of these data, we chose 5 minutes as the standard exposure duration of MDCK-London cells with CL Buffer for subsequent experiments; we reasoned that C_q_ variability among experimental replicates can be reduced by avoiding the steeper portion of the RNA yield curve entailed by harvesting of lysate at earlier than 5 minutes.

Upon finalizing the formulation for CL Buffer, we assessed the efficiency of our RT-qPCR targeting the matrix gene of influenza virus. A standard, consisting of purified total RNA from MDCK-London cells infected with influenza virus, was serially diluted. In order to enhance comparability with experimental samples, the diluent for the RNA standard was a lysate of uninfected MDCK-London cells prepared using CL Buffer (10 mM Tris pH 7.4, 0.25% Igepal CA-630, and 150 mM NaCl; cell exposure for 5 minutes at room temperature). One μL of each dilution was subjected to one-step RT-qPCR (10 μL total volume). PCR efficiency was typically ~100%, and linearity was observed over at least a 5 log_10_ range (Supplementary Fig. 1). Melt curve analysis revealed a single major peak at 81.5°C. A similar dilutional analysis was also performed using a commercially available synthetic exogenous RNA spike reagent (Supplementary Fig. 2); the results suggest an absence of meaningful levels of RT-qPCR inhibitors under our experimental conditions.

**Figure 3:**
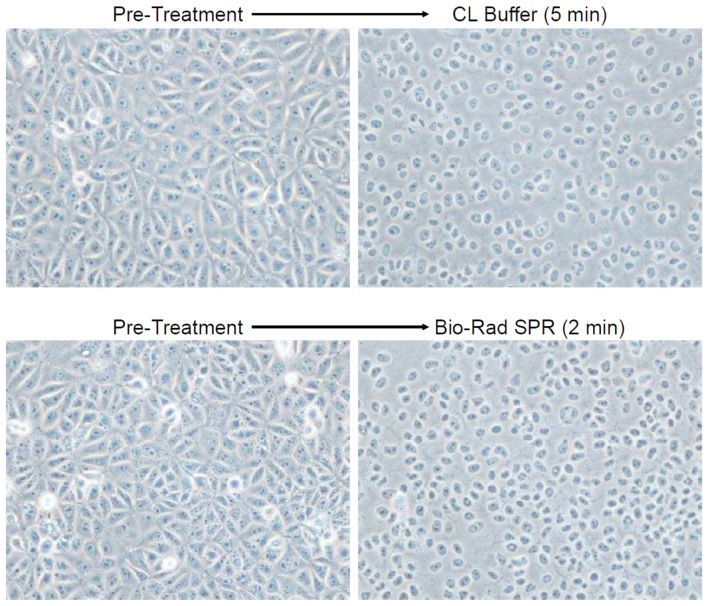
Morphology of cells exposed to cell-lysis reagent. Phase-contrast micrographs of MDCK-London cells in 24-well culture plate wells (300,000 cells/well; 6 hours after seeding) are shown before and after treatment (identical fields) with either CL Buffer or Bio-Rad SPR.

MDCK-London cells exposed to CL Buffer were observed microscopically in order to gain insight on the cell-lysis process. Identical micrographic fields of cells before and after treatment, either with CL Buffer or Bio-Rad SPR, are shown in Fig. 3. After exposure to CL Buffer, cell borders became less distinct and cell nuclei appeared condensed; it is notable, however, that cell nuclei remained adherent and fixed in position. Thus, the morphology of the cell-monolayer remnant after exposure to CL Buffer suggests that lysis/permeabilization occurred in a gentle manner. Cells exposed to CL Buffer (5 minutes) were similar in appearance to cells exposed to Bio-Rad SPR (2 minutes).

### Analysis of cell lysates subjected to stress by incubation at 37°C

We next subjected cell lysates to various stresses to assess RNA stability and impact on RT-qPCR. MDCK-London cells (24-well plate; 300,000 cells per well) were infected with influenza virus; six hours post-infection, cell monolayers were washed once with PBS and then exposed to 200 μL of CL Buffer or Bio-Rad SPR. Freshly prepared lysates were collected in microfuge tubes and immediately placed in a 37°C incubator for up to 4 hours. Following exposure to stress at 37°C, 1 μL of each resulting lysate was analyzed directly by one-step RT-qPCR targeting the influenza virus matrix gene; in parallel, total RNA was column-purified (Qiagen RNeasy) immediately from the remainder of the lysates and analyzed with the Experion system. Representative results are shown in Supplementary Fig. 3. RNA in lysates generated with CL Buffer was found to be surprisingly stable, with RQIs generally above 9.0 even following 1–2 hours at 37°C; for the experiment shown, RQIs decreased below 9.0 (to 8.7) after 4 hours at 37°C (Supplementary Fig. 3a). Associated C_q_ values were minimally affected by the limited RNA degradation. Mean C_q_ across all conditions was 17.62 (range: 17.06 to 18.54). Similar results were obtained with samples prepared using Bio-Rad SPR (Supplementary Fig. 3b).

Samples were also assessed for stability under frozen storage at −20°C or −80°C. Prior to analysis, samples underwent one to three cycles of freeze/thaw. Samples were subjected to RT-qPCR and Experion analysis (following RNA purification) as described above. After 18 days, lysates generated with CL Buffer and stored at −20°C exhibited modest RNA degradation (RQIs of 8.9–9.7; Supplementary Fig. 4a), which appeared to be prevented either by storage at −80°C (Supplementary Fig. 4a) or addition of exogenous RNase inhibitor (1 unit/μL) to CL Buffer (Supplementary Fig. 4b). C_q_ values of lysates generated with CL Buffer (in the absence or presence of exogenous RNase inhibitor) were minimally impacted by the experimental conditions, and compared favorably with those of lysates generated with Bio-Rad SPR (Supplementary Fig. 4c).

**Figure 4:**
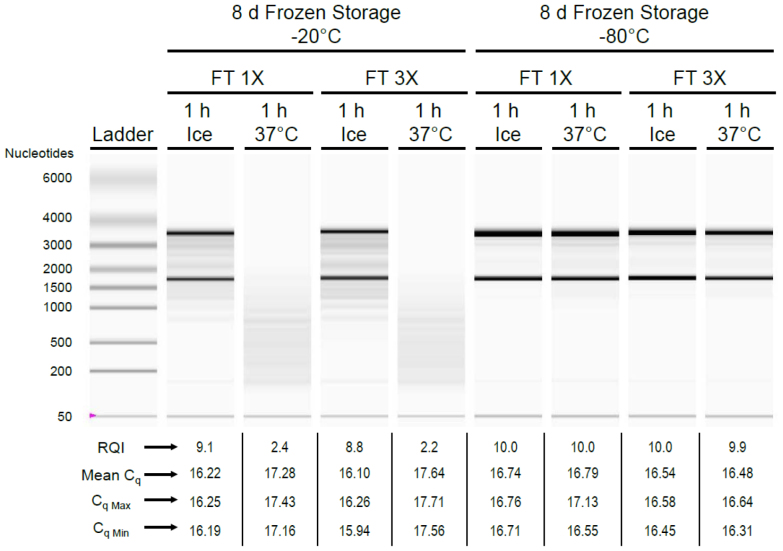
Cell-lysate RNA stability following frozen storage and stress at 37°C. Cell lysates (200 μL) were prepared from MDCK-London cells (300,000/well; 24-well plate) infected with influenza virus (10,000 TCID_50_/well) by exposing them to CL Buffer for 5 min at ~22°C. Lysates were stored frozen at −20°C or −80°C for 8 days. During storage, some of the lysates experienced two intervening cycles of freeze/thaw by thawing at room temperature and then immediately replacing in the freezer (no more than one freeze/thaw cycle per 24 hour period). Following 8 days of frozen storage, samples were thawed (total freeze/thaw: 1× or 3×) and either placed on ice or in a 37°C incubator for 1 hour. One μL of each lysate was analyzed directly by one-step SYBR Green RT-qPCR with primers targeting the influenza virus matrix gene; total RNA was purified immediately from the remaining lysates and subjected to microfluidics-based electrophoresis using the Bio-Rad Experion system. Virtual gel image, sample RNA yields, and RNA Quality Indicators (RQIs) are shown; associated mean C_q_ values as well as C_q Max_ and C_q Min_ values from RT-qPCR (n = 3) are also indicated.

Finally, we chose to assess stresses more realistically modeled after those associated with routine handling of experimental samples, *i.e*., a combination of frozen storage (8 days at −20°C or −80°C) with freeze/thaw cycles (1× or 3×) followed by incubation either on ice or at 37°C for 1 hour (Fig. 4). For lysate samples initially stored frozen at −20°C, extensive RNA degradation was observed after post-thaw incubation for 1 hour at 37°C *vs*. on ice (RQIs decreased 9.1 → 2.4 and 8.8 → 2.2 for samples experiencing freeze/thaw 1× or 3×, respectively); associated C_q_ values correspondingly shifted (16.22 → 17.28 and 16.10 → 17.64 for samples experiencing freeze/thaw 1× or 3×, respectively) in response to 37°C stress, corroborating the notion that deterioration in sample RNA quality can impair the accuracy of RT-qPCR^10^. Samples stored at −80°C behaved in a starkly different manner. RNA quality was preserved for samples stored at −80°C even after post-thaw 37°C exposure for 1 hour (RQI: 9.9–10.0), reminiscent of freshly prepared lysate (Supplementary Fig. 3a); C_q_ values were similar across temperature stress conditions (mean C_q_: 16.48–16.79), suggesting that post-thaw 37°C exposure for 1 hour did not influence RT-qPCR results substantially for samples stored at −80°C. In addition, freeze/thaw cycles experienced by samples during −80°C storage (1× *vs*. 3×) had no impact on RNA quality or RT-qPCR.

### Application of cell-lysate approach to assess influenza virus neutralization

**Figure 5:**
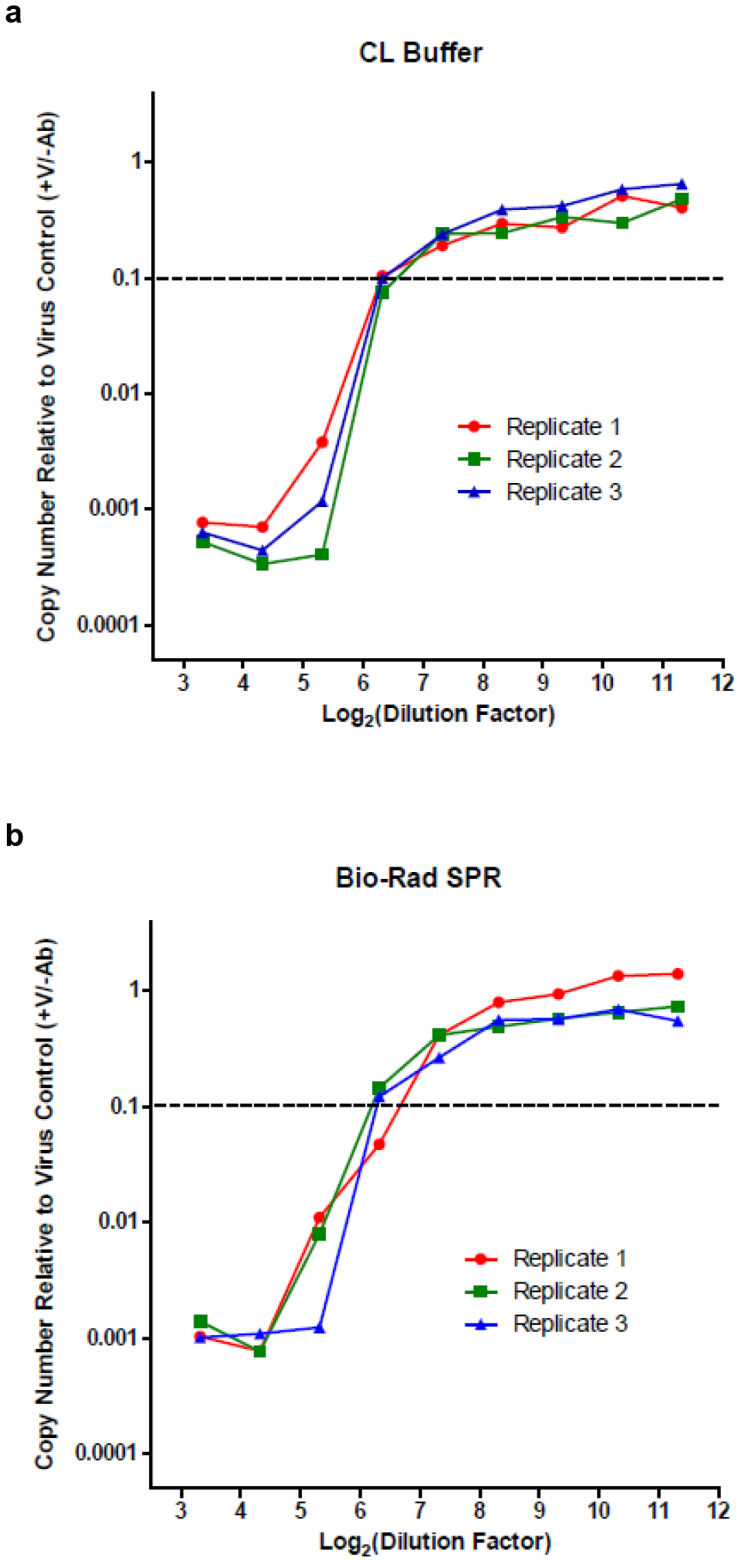
RT-qPCR-based microneutralization of influenza virus. In a well of a 96-well culture plate, influenza virus (A/Brisbane/59/2007; 1000 TCID_50_) was mixed with a dilution from a 2-fold dilution series generated using a human serum sample with specific neutralizing activity. After an incubation for 1 hour at 37°C, trypsinized MDCK-London cells (30,000 per well) were added. At 6 hours post-infection, cell lysates were prepared using (a) CL Buffer or (b) Bio-Rad SPR and subjected to RT-qPCR (a single reaction per original culture well). RNA copy numbers were normalized to the mean value obtained from infected wells in the absence of neutralizing serum (virus control wells; +V/−Ab). The neutralization titer was defined as the reciprocal of the highest dilution factor of serum necessary to inhibit the PCR signal by 90% (threshold indicated by dotted line). Each serum dilution was assessed in triplicate infections; wells consisting of a replicate serum dilution series (corresponding to a row of wells in the original culture plate) are shown independently.

We ultimately applied CL Buffer to our originally intended use to measure virus neutralization. As described earlier^1^, influenza virus inoculum (1000 TCID_50_ of A/Brisbane/59/2007) was mixed with an equal volume from a serum dilution series in individual wells of a 96-well culture plate and incubated for 1 hour at 37°C. A suspension of MDCK-London cells (30,000 per well) was subsequently added and the plate was incubated at 37°C for an additional 6 hours (by which time the cells had adhered). Cell lysates were then prepared using either CL Buffer or Bio-Rad SPR and subjected to RT-qPCR analysis with primers targeting the influenza virus matrix gene. The results are shown in Fig. 5. Data are normalized to mean values in control wells in the absence of neutralizing serum. Neutralization was assessed in triplicate, and individual neutralization curves (representing independent rows of the 96-well plate) are presented. Results obtained with CL Buffer (Fig. 5a) were similar to those obtained with Bio-Rad SPR (Fig. 5b). Each curve crossed the pre-specified neutralization threshold (10% of the mean value observed in virus-infected wells in the absence of neutralizing serum) at a consistent dilution of serum (within 2-fold across replicates; geometric mean titer of 63 and 50 for CL Buffer and Bio-Rad SPR, respectively).

## Discussion

We previously described an approach to measure virus neutralization using an assessment based on RT-qPCR^1^,^2^. We were able to avoid sample nucleic acid extraction and purification, hitherto considered to be critical rate-limiting steps for conventional PCR methodologies, by using a commercially available reagent (Bio-Rad SPR) that generates RT-qPCR-ready cell lysates with minimal manipulations. Similar reagents are now available from a number of sources (Ambion Cells-to-C_T_, Invitrogen CellsDirect, Roche RealTime Ready Cell Lysis, etc.). The ease of use associated with these cell-lysis reagents offers considerable promise in expanding the scope of applications for RT-qPCR, particularly in high-throughput settings requiring compatibility with automation. Recent studies have demonstrated that use of crude cell lysate (prepared using commercial reagents) as direct input for RT-qPCR requires minimal compromise in terms of accuracy compared with a traditional workflow involving RNA purification^5^,^6^. Indeed, the cell-lysate approach was found to be associated with superior sensitivity in some cases^5^.

However, cost connected with these commercially available cell-lysis reagents is not trivial. As described^1^,^2^, our RT-qPCR-based virus microneutralization assays currently cost ~$1 per well of a 96-well plate, consisting of ~$0.60 for Bio-Rad SPR (100 μL) and ~$0.40 for the one-step RT-qPCR kit (10 μL reaction volume). Thus, in spite of the technical virtues inherent in using cell lysates for direct analysis by RT-qPCR, cost can be a hindrance to throughput, depending on the scale of endeavor, if one were to rely exclusively on commercial reagents for cell-lysate preparation.

In the present study, we demonstrate that a simple buffer containing a non-ionic detergent can generate cell lysates amenable to downstream RT-qPCR. CL Buffer is associated with negligible cost on a per-sample basis. RT-qPCR results obtained with independent cell-culture replicates using CL Buffer were highly consistent and comparable with those obtained using Bio-Rad SPR. Total RNA in lysates freshly generated with CL Buffer appears to be surprisingly resistant to degradation even in the absence of exogenous RNase inhibitor as a buffer component. Furthermore, resistance to RNA degradation can be preserved provided that lysate samples are frozen at −80°C. The mammalian RNase inhibitor, a ubiquitously expressed cytosolic protein, is known to be somewhat labile^11^,^12^. We hypothesize that sample RNA stability may be due to endogenous RNase inhibitor present in the lysate whose activity is maintained at −80°C storage. Circumventing the need to add exogenous RNase inhibitor from a commercial source is notable in terms of logistics, as its addition to a lysis buffer at the typically recommended concentration (1 unit/μL) would entail a considerable expense (~$3 per 100 μL of buffer).

Others have also recently reported cell-lysis reagent formulations for the purpose of direct analysis by RT-qPCR. One example by Ho *et al*. uses non-ionic detergents (both Triton X-100 and NP40), but their buffer formulation differs from our own in that a commercial inhibitor of RNase is included (Ambion RNAsecure)^6^. Another example by Svec *et al*. uses a simple solution of bovine serum albumin (BSA; 1 mg/mL) in pure water for directly lysing fewer than five hundred cells^13^; the mechanism of cell lysis is presumably hypotonic stress. Interestingly, addition of exogenous RNase inhibitors was found to be unnecessary when using BSA/water for cell lysis. Although the investigators concluded that RNase-mediated RNA degradation may be minimal under their experimental conditions on the basis of the observed dispensability of exogenous RNase inhibitors, an alternative explanation (consistent with our own) is that endogenous RNase inhibitor activity may be maintained by the sample storage temperature used for this study (−80°C). The studies by Ho *et al*. and Svec *et al*. did not include a direct assessment of sample RNA quality, which was perhaps precluded by the micro-scale nature of their samples. Nevertheless, these studies, as well as our own, corroborate the general feasibility of using cell lysates generated with non-commercial cell-lysis reagents for gene-expression analysis.

Proof-of-concept for our cell-lysate approach was established using a derivative of MDCK cells infected with influenza virus. CL Buffer can be used as a substitute for Bio-Rad SPR in our previously described microneutralization assay for influenza virus^1^. Other related high-throughput applications using CL Buffer and MDCK cells include RT-qPCR-based library screening for chemicals or RNAi species that can inhibit influenza virus replication for the purpose of identifying lead compounds in the development of antivirals. Future studies are aimed at assessing the generalizability of our approach in terms of cells and gene targets.

## Methods

### Cell culture

MDCK-London cells^14^ were propagated using DMEM (Mediatech, Inc.) supplemented with 10% fetal bovine serum (Hyclone) and 2 mM glutamine. Infection with influenza virus (A/Brisbane/59/2007) was performed in an infection medium consisting of DMEM supplemented with glutamine (2 mM), HEPES (25 mM), bovine serum albumin (0.2%; A7888; Sigma), and TPCK-trypsin (1 μg/mL; T1426; Sigma). For experiments using a 24-well cell-culture plate format, a suspension of trypsinized cells (thoroughly washed to remove serum; 300,000 cells/well) was mixed with virus (10,000 TCID_50_/well) in infection medium (1 mL/well). Cells were allowed to adhere and cell lysates were prepared at 6 hours post-infection.

### Preparation of RT-qPCR-ready cell lysates

MDCK-London cells in 24-well plates were washed once with PBS (1 mL/well). Cell lysates were prepared by exposing cell monolayers to 200 μL/well of Bio-Rad iScript Sample Preparation Reagent (referred to as Bio-Rad SPR; 170-8898) or Cell-Lysis (CL) Buffer. The final formulation of CL Buffer consisted of 10 mM Tris-HCl pH 7.4, 0.25% Igepal CA-630, and 150 mM NaCl. CL Buffer was freshly prepared from the following stock solutions on the day of experimentation: 1 M Tris-HCl (T2194; Sigma), 10% Igepal CA-630 (I8896; Sigma); and 5 M NaCl (351-036-100; Quality Biological, Inc.). All reagents were molecular biology grade and dilutions were made with DEPC-treated water (351-068-721; Quality Biological, Inc.). For certain experiments, CL Buffer also included MgCl_2_ (M1028; Sigma) or RNasin Plus RNase Inhibitor (N2615; Promega). Both Bio-Rad SPR and CL Buffer were equilibrated to room temperature prior to use. Cells were exposed for the indicated times (typically 2 min for Bio-Rad SPR and 5 min for CL Buffer). The resulting lysates were carefully collected without disturbing the cell-monolayer remnants and either analyzed immediately or stored frozen (−20°C or −80°C).

### RT-qPCR analysis of influenza virus matrix gene expression

Experiments were designed to be compliant with MIQE guidelines^15^. RT-qPCR analysis was performed as described in an earlier study^1^. PCR primers (forward: AAGACCAATCCTGTCACCTCTGA; reverse: CAAAGCGTCTACGCTGCAGTCC) amplifying a highly conserved 104 bp region of the matrix gene of influenza A viruses^16^ were used in one-step SYBR Green RT-qPCR. Each reaction contained: template (1 μL of cell lysate), 1× iScript One-Step SYBR Green RT-PCR Supermix (170–8893; Bio-Rad), 600 nM of each primer (synthesized at the Facility for Biotechnology Resources; CBER, FDA; Bethesda, MD), and nuclease-free water to 10 μL. A CFX96 real-time PCR instrument (Bio-Rad) was used with the following protocol: 50°C for 10 min (1×), 95°C for 5 min (1×), 95°C for 10 sec/61°C for 15 sec/72°C for 30 sec (40×); data collection occurred after the 72°C extension step. Total RNA purified from MDCK-London cells infected with the influenza virus strain A/PR/8/34 was used as an RT-qPCR quantification standard as described previously^1^. For each RT-qPCR run, a 10-fold dilution series of the standard (using cell lysate prepared from uninfected cells as the diluent) was assessed in at least duplicate in order to validate RT-qPCR performance and facilitate quantification. In addition, each RT-qPCR run included negative controls (uninfected lysate as input) and no-reverse transcription controls (initial dilution of the RNA standard described above as input); these controls typically result in no amplification or low-level non-specific amplifications (suggested by melt curve analysis) with C_q_'s > 36. It is important to note that there are no DNA intermediates in the life cycle of influenza virus.

### Microfluidics-based assessment of RNA

Total RNA from cell lysates was purified using the RNeasy Mini kit (Qiagen) according to the “cleanup” protocol supplied with the kit. Starting with ~200 μL of cell lysate, 700 μL of Buffer RLT and 500 μL of ethanol were added; the mixture was passed through an RNeasy Mini spin column. Following the prescribed washing steps, purified RNA was eluted in 30 μL of nuclease-free water and stored at −80°C until assessment. Purified RNA samples (1 μL) were subjected to microfluidics-based Experion RNA StdSens electrophoresis analysis (Bio-Rad) according to the system manufacturer's protocol. Sample RNA concentrations, as measured by Experion, typically ranged from 100–300 ng/μL (within the recommended concentration range of 5–500 ng/μL for the StdSens chip).

### RT-qPCR-based microneutralization assay for influenza virus

Influenza virus microneutralization was performed as described previously^1^. A sample of human serum^17^ from an individual with a history of influenza vaccination was acquired from W. Wang and C. Weiss (Division of Viral Products, CBER, FDA). The serum sample was obtained with written informed consent following ethics approval by the Research Involving Human Subjects Committee (RIHSC) at the US Food and Drug Administration. The serum sample was heat-inactivated by incubation at 56°C for 30 minutes prior to use. Neutralization experiments were performed using infection medium. Virus inoculum (1000 TCID_50_/50 μL) was mixed with a dilution of serum (50 μL) in a well of a 96-well culture plate. Following an incubation for 1 hour at 37°C, a suspension of MDCK-London cells (30,000 cells/100 μL) was added. At 6 hours post-infection, cell lysates were prepared by washing the cells once with PBS and adding 100 μL/well of Bio-Rad SPR or CL Buffer equilibrated to room temperature; cells were exposed for 2 minutes (Bio-Rad SPR) or 5 minutes (CL Buffer). Lysates were collected and stored frozen at −20°C (Bio-Rad SPR samples) or −80°C (CL Buffer samples) until assessment. Virus replication, as reflected by matrix gene expression, was quantified by subjecting lysates (1 μL) to analysis by RT-qPCR. RNA quantity in each experimental sample was normalized against the mean value (n ≥ 3) obtained from control wells in which cells were infected in the absence of neutralizing serum (virus control wells). Neutralization titer was determined as the reciprocal of the highest serum dilution (prior to the addition of virus or cells) resulting in at least 90% inhibition of the RT-qPCR signal.

## Author Contributions

K.S., B.T. and H.M. were responsible for design and execution of experiments. H.M. supervised the study and drafted the manuscript. All authors reviewed and revised the manuscript.

## Supplementary Material

Supplementary Information Supplementary Information
