# Supplementary material for: A simple, inexpensive method for preparing cell lysates suitable for downstream reverse transcription quantitative PCR
Source: Sci Rep. 2014 Apr 11;4:4659. doi: 10.1038/srep04659 (PMC3983595; doi:10.1038/srep04659)
Supplement: Supplementary Information [file srep04659-s1.pdf]

A simple, inexpensive method for preparing cell lysates suitable for downstream reverse transcription quantitative PCR

Kenneth Shatzkes[†], Belete Teferedegne[†], and Haruhiko Murata*

[†]These authors contributed equally to this work.

*Correspondence and requests for materials should be addressed to H.M. (haruhiko.murata@fda.hhs.gov).

Supplementary Table 1
Assessment of $MgCl_2$
(10 mM Tris pH 7.4; 0.25% Igepal CA630; 150 mM NaCl; 0, 1.5, or 5 mM $MgCl_2$)

$MgCl_2$ (mM)	Exp 1		Exp 2		Exp 3	
	Mean C_q [a]	C_q Range[a,b]	Mean C_q [a]	C_q Range[a,b]	Mean C_q [a]	C_q Range[a,b]
**0**	18.96	0.47	19.95	0.94	19.68	0.73
**1.5**	20.14	0.01	21.79	0.58	21.22	0.27
**5**	20.90	0.62	22.39	0.37	21.29	0.34

[a] Calculated from three RT-qPCR replicates
[b] $C_{q\ Max} – C_{q\ Min}$

Supplementary Table 2
Assessment of pH
(10 mM Tris pH 7.0, 7.4, or 8.0; 0.25% Igepal CA630; 150 mM NaCl)

pH	Exp 1		Exp 2	
	Mean C_q [a]	C_q Range[a,b]	Mean C_q [a]	C_q Range[a,b]
**7.0**	19.27	0.40	19.69	1.35
**7.4**	18.85	0.35	19.68[c]	0.73[c]
**8.0**	18.85	0.44	19.54	0.91

[a] Calculated from three RT-qPCR replicates
[b] $C_{q\,Max} - C_{q\,Min}$
[c] Data also represented in Supplementary Table 1 (Experiment 3; 0 mM $MgCl_2$)

Supplementary Figure 1. *Efficiency of one-step SYBR Green RT-qPCR targeting the matrix gene of influenza virus*

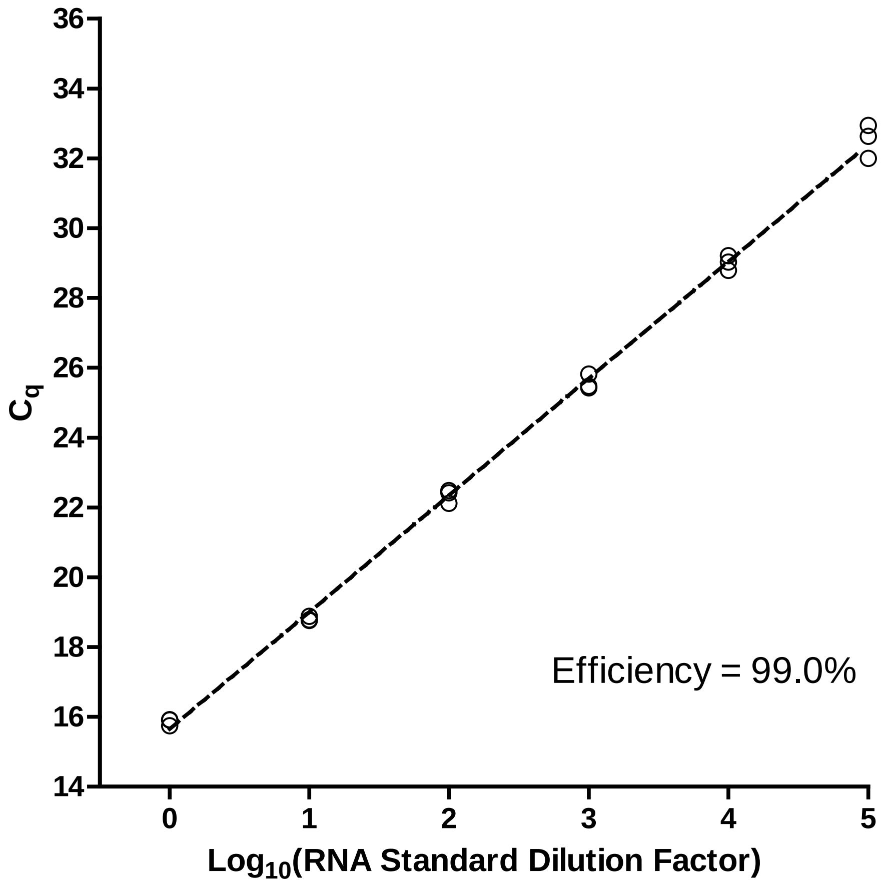

Total RNA was purified from MDCK-London cells infected with A/PR/8/34 and used as a quantification standard for this study. The RNA standard was serially diluted using lysate from uninfected MDCK-London cells (prepared with CL Buffer) as the diluent. The initial dilution contained ~10 ng of standard RNA per µL. One µL of each dilution was subjected to one-step SYBR Green RT-qPCR (10 µL total volume) with primers targeting the influenza virus matrix gene. Each dilution was assessed in triplicate. Quantification cycle (C_q) is plotted against $\log_{10}$(RNA standard dilution factor).

Supplementary Figure 2. *One-step SYBR Green RT-qPCR targeting a synthetic exogenous RNA spike control reagent*

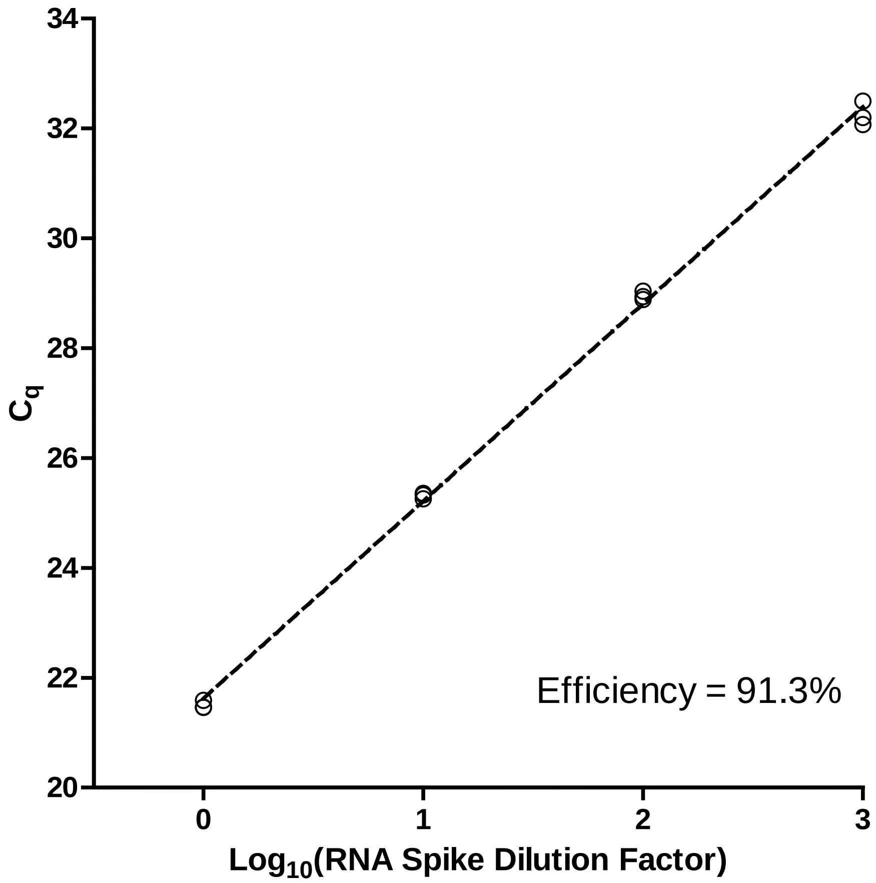

A commercially available synthetic RNA spike control was obtained (Solaris RNA Spike Control; K-002200-C1; Thermo Scientific). The RNA spike control was serially diluted using lysate from uninfected MDCK-London cells (prepared with CL Buffer) as the diluent. The RNA spike reagent was supplied at 100X concentration; the initial dilution (1:10) was tested at 10X concentration. One µL of each dilution was subjected to one-step SYBR Green RT-qPCR (10 µL total volume) with primers targeting the spike RNA (forward primer TGCAAAGCCAATTCCCGAAG; reverse primer CCATTGTAGTGAACAGTAGGAC; sequences provided by the RNA spike supplier). Each reaction contained: template (1 µL of RNA dilution), 1X iScript One-Step SYBR Green RT-PCR Supermix (170–8893; Bio-Rad), 100 nM of each primer, and nuclease-free water to 10 µL. A CFX96 real-time PCR instrument (Bio-Rad) was used with the following protocol: 50°C for 10 min (1X), 95°C for 5 min (1X), 95°C for 10 sec/60°C for 30 sec (40X). Each dilution was assessed in triplicate. Quantification cycle (C_q) is plotted against $\log_{10}$(RNA spike dilution factor). Melt curve analysis of reactions containing detectable quantities of the RNA spike revealed a single peak at ~78°C. Negative controls (uninfected lysate as input) and no-reverse transcription controls (10X RNA spike as input) yielded non-specific amplifications with C_q's > 36.

Supplementary Figure 3. *RNA stability of freshly prepared cell lysates stressed at 37°C*

a

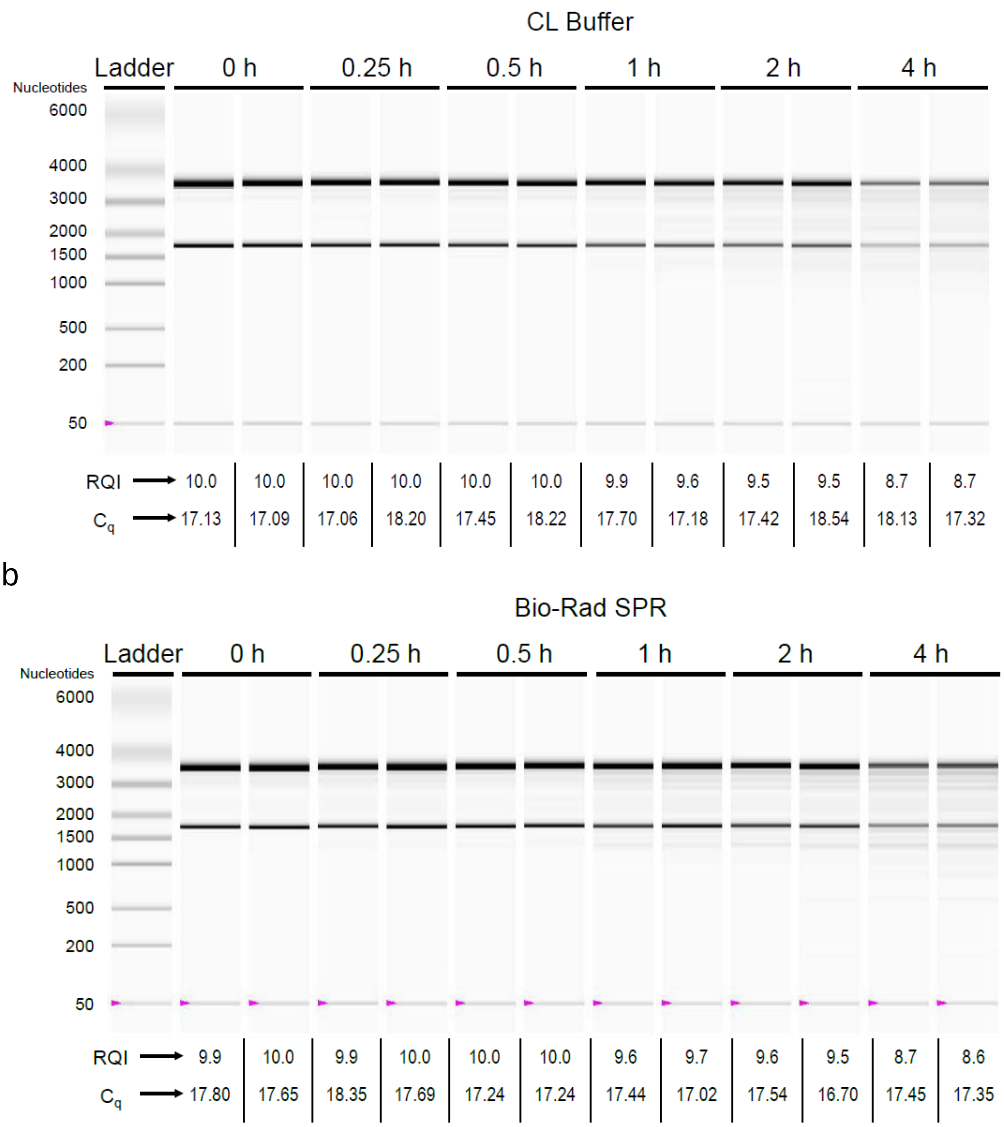

Cell lysates (200 µL) were prepared from MDCK-London cells (24-well plate; 300,000/well) infected with influenza virus (10,000 $TCID_{50}$/well) by exposing them to (a) CL Buffer (5 min) or (b) Bio-Rad SPR (2 min). Lysates were then placed in a 37°C incubator for up to 4 hour. Following stress, 1 µL of each lysate was analyzed directly by one-step SYBR Green RT-qPCR with primers targeting the influenza virus matrix gene; total RNA was purified immediately from the remaining lysates and subjected to microfluidics-based electrophoresis using the Bio-Rad Experion system. Virtual gel images, sample RNA yields, and RNA Quality Indicators (RQIs) are shown; associated C_q values from RT-qPCR are also indicated.

Supplementary Fig. 4. *RNA stability of cell lysates stored frozen at -20°C or -80°C*

a

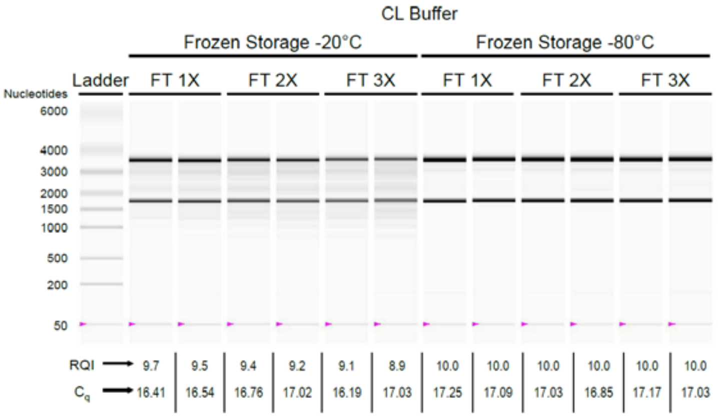

b

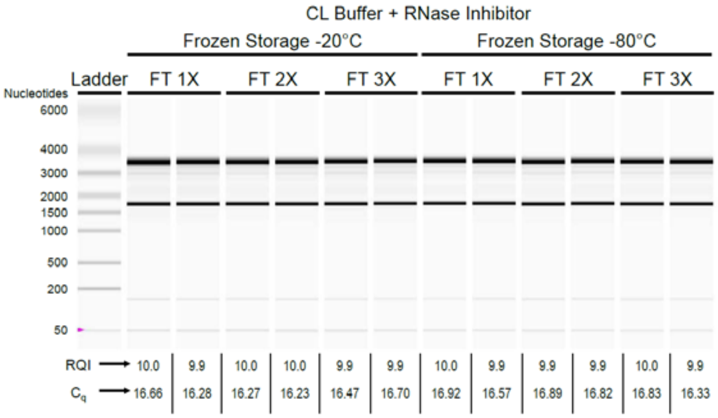

c

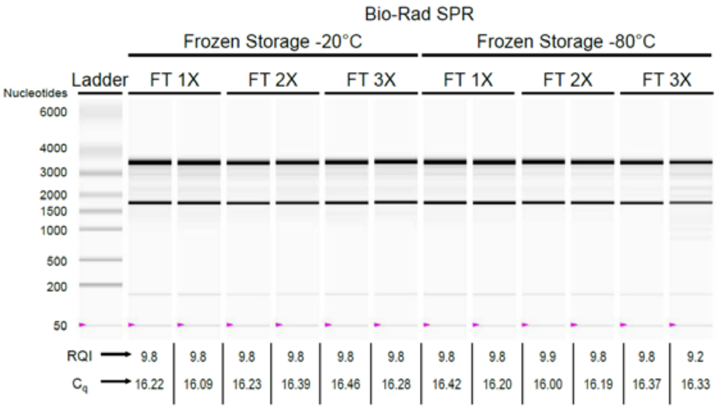

Cell lysates (200 µL) were prepared from MDCK-London cells (24-well plate; 300,000/well) infected with influenza virus (10,000 $TCID_{50}$/well) by exposing them to (a) CL Buffer (5 min), (b) CL Buffer supplemented with 1 unit/µL RNasin Plus RNase Inhibitor (5 min), or (c) Bio-Rad SPR (2 min). Lysates were stored frozen at -20°C or -80°C for 18 days. During storage, lysates experienced cycles of freeze/thaw by thawing at room temperature and then immediately replacing in the freezer (no samples

experienced more than one freeze/thaw cycle per 24 hour period). Following frozen storage, samples were thawed (total freeze/thaw: 1X, 2X, or 3X) and 1 µL of each lysate was analyzed directly by one-step SYBR Green RT-qPCR with primers targeting the influenza virus matrix gene; total RNA was purified immediately from the remaining lysates and subjected to microfluidics-based electrophoresis using the Bio-Rad Experion system. Virtual gel images, sample RNA yields, and RNA Quality Indicators (RQIs) are shown; associated C_q values from RT-qPCR are also indicated.
